# Pedicled Anterior Lateral Thigh Flap in Managing a Bilateral Groin Contracture

**DOI:** 10.1155/2014/451356

**Published:** 2014-09-22

**Authors:** Ferdinand Nangole, Peter Biribwa, Stanley Khainga

**Affiliations:** Department of Surgery, University of Nairobi, P.O. Box 2212, Nairobi 00202, Kenya

## Abstract

A fifteen-year-old female patient presented with a severe bilateral groin contracture for the last 8 years. She had sustained burns at the age of seven years. Three attempts to release the contracture with split thickness skin grafts had been done without success. A pedicled anterior lateral thigh flap was raised and advanced into the defect after the contracture had been released. Postoperatively the patient healed well without any complications and was able to achieve hip abduction of about 130 degrees.

## 1. Introduction

Postburn groin contractures can result in severe morbidity and functional impairment to the patient. Severe contractures result in limitation of movement at the hip joints affecting the normal gait of the patient. Due to limited abduction at the hip joints these patients especially if females would have difficulties in passing stool and urine. They would also experience poor perineal hygiene with increased possibilities of urinary tract infections. The contractures would further interfere with normal sexual functions and may prevent normal deliveries.

Releasing of the contractures could be achieved by either skin graft or flap surgery. Most of the flaps utilized are local flaps such as z plasties [1]. This may however not be possible in cases where there are wide and extensive scarred tissues. Skin grafts on the other hand are associated with high recurrence rates and poor graft take [2]. Regional, distant, or free flaps may thus be the only options in cases where there are extensive and severe contractures [1–3].

## 2. Case Presentation

A fifteen-year-old patient presented to us with a seven-year history of contractures involving the groin bilaterally. The contractures were secondary to burns. She had sustained burns after hot water spillage at the age of eight years. In her past medical history she had had three attempts of contracture release with split thickness skin grafts. These were however not successful.

**Figure 1:**
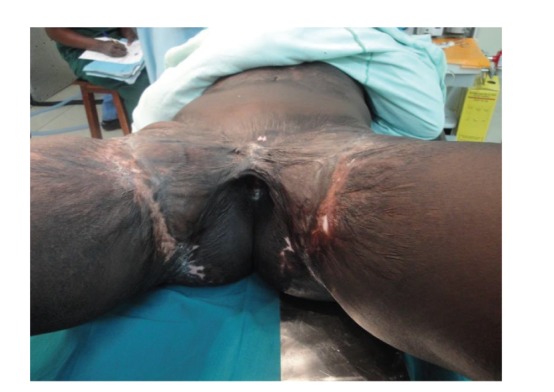
Inferior view of the patient with bilateral contractures.

**Figure 2:**
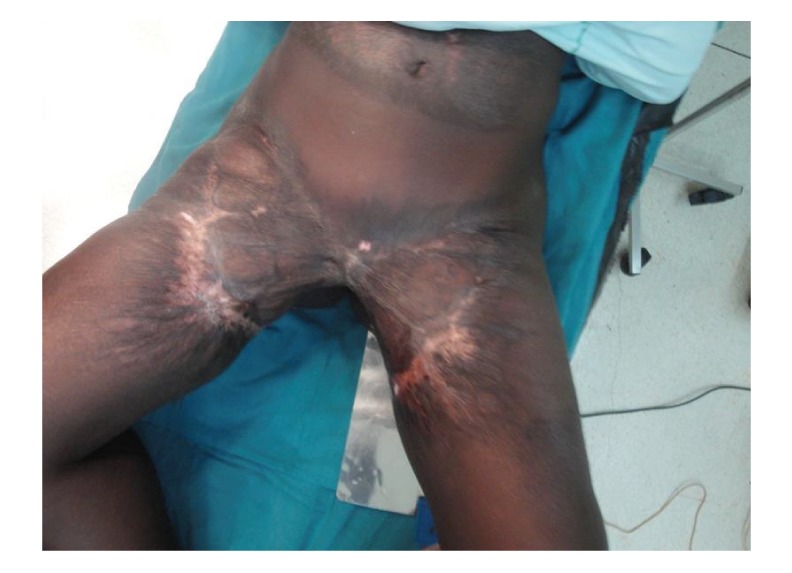
Anterior view of the patient with bilateral groin contracture.

On examination she was in good general condition. She had low self-esteem and had stained inner clothing with urine. She had poor perineal and vulva hygiene. She had multiple and extensive scarring in the pubic and groin region bilaterally. There was evidence of split thickness graft donor site from both thighs. She had hip abduction of about 40 degrees (Figures 1 and 2). She also had a slow and limited gait with a flexion deformity at the pelvis. All other systems were normal.

**Figure 3:**
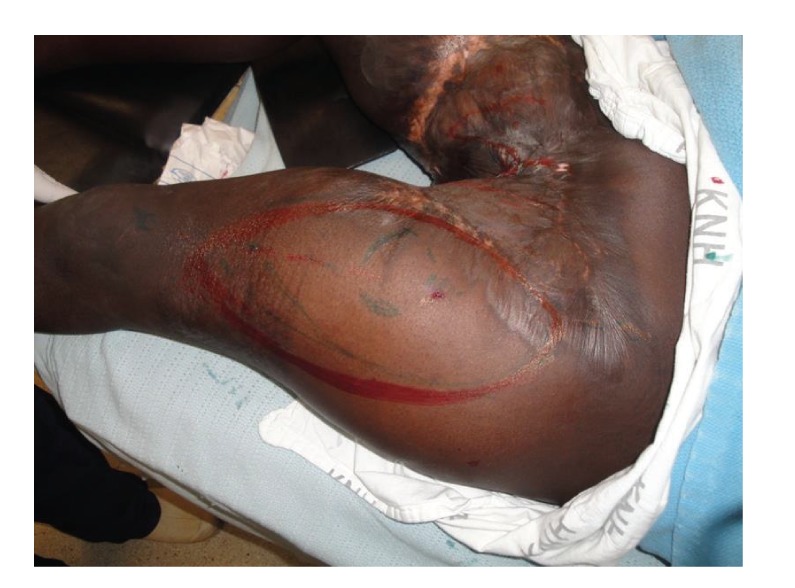
Marking of the left anterior lateral thigh flap to be raised with an inner green circle indicating the location of the perforator.

**Figure 4:**
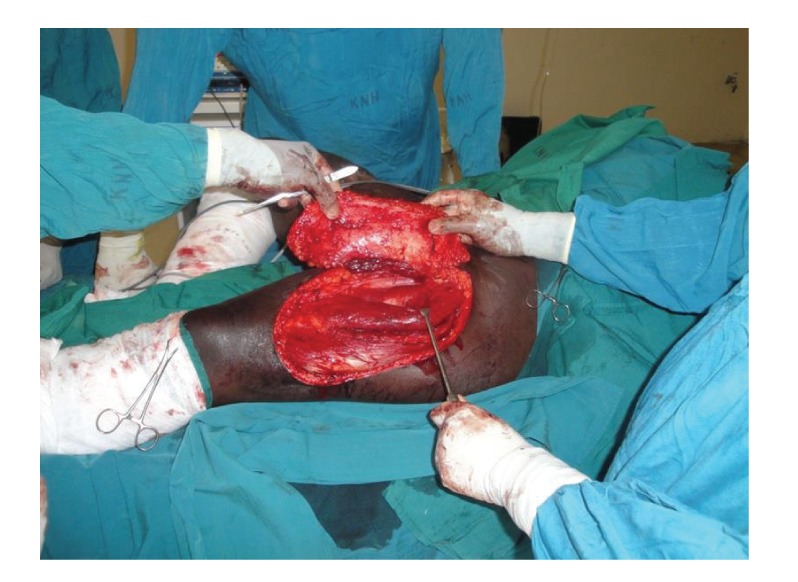
Anterior lateral thigh flap being raised from the surrounding tissues.

**Figure 5:**
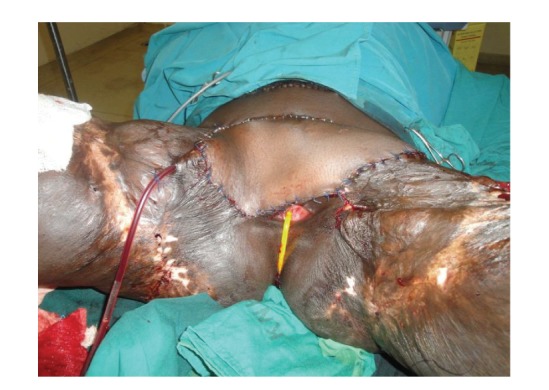
Anterior lateral thigh flap fully covers both groins after release of the contractures.

After evaluation, the patient was booked for contracture release and reconstruction with a pedicled anterior lateral thigh flap. The contracture was released bilaterally and all the scarred tissues removed. A suitable perforator was identified with handheld ultrasound Doppler. This was roughly at the midpoint on a line from the anterior superior iliac spine and the patella (Figure 3). The flap was raised based on the perforator after careful dissection from the surrounding muscle (Figure 4). The plane of dissection was in the subfascial plane over the vastus lateralis and the rectus femoris muscles. The pedicle was progressively dissected from distal to proximal up to the lateral circumflex vessels. The flap was then advanced into the defect and a drain was inserted beneath the flap (Figure 5). The recipient site was closed with a split thickness skin graft harvested from the contralateral thigh.

**Figure 6:**
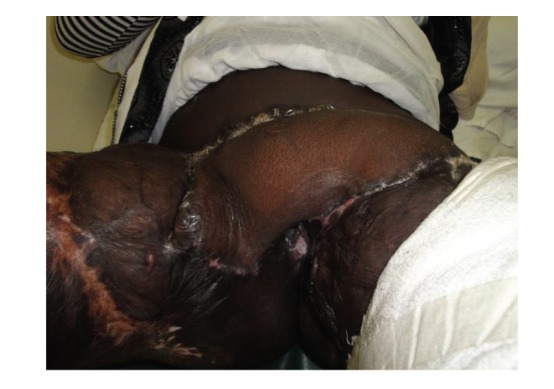
Postoperative view of the patient at 1 month after surgery.

**Figure 7:**
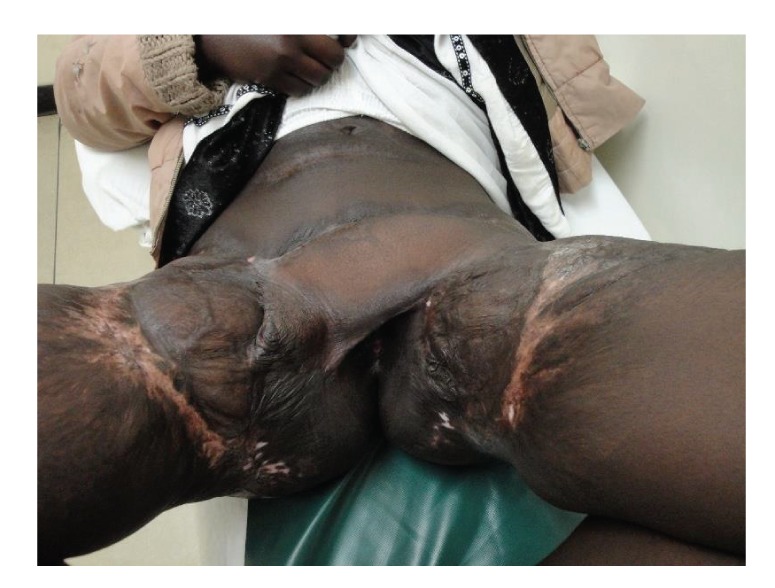
Postoperative view of the patient at one year after surgery.

Postoperatively the patient did well with no complications (Figure 6). At one year of follow-up, there was no recurrence of the contracture. Her hip abduction had improved to about 130 degrees (Figure 7). Her gait had also improved and had a better self-esteem. Her perineal hygiene had also improved and she was able to pass urine and stool well.

## 3. Discussion

Bilateral postburn groin contractures may present with severe and disabling morbidity to the patient. Prolonged contractures may result in skeletal deformities such as posterior dislocation of the hip with limb shortening. Prompt and adequate release of the contractures should thus be the aim of the team managing such patients.

Though skin grafts have been used extensively in managing contractures, the groin region offers further challenges. This region is a difficult area to immobilize postoperatively, resulting in poor skin graft take and high contracture recurrence [1, 2]. This is evidenced by our patient who had had three sessions of skin grafts with contracture recurrences.

Local advancement flaps such as Z palsies, Y to V flaps, or even the seven flap plasty have been reported in the literature as being effective in the management of groin contractures [3]. They are however limited to linear contractures with normal tissues around the contractures. Our patient had extensive scarring both medially and inferiorly, extending to the perineum and the anterior abdominal wall making it difficult to use any local advancement flaps.

Regional flaps that could have been used in our patient other than the anterior lateral thigh flap include the inferiorly based rectus abdominis myocutaneous flap. The donor site morbidity with this flap is however likely to be worse than that of the anterior lateral thigh flap. The morbidity includes anterior abdominal wall weaknesses as well as extensive scarring on the abdomen. The groin flap or the anterior medial thigh flaps were not possible options in our patient due to the severe and extensive scarring both anteriorly and medially. Posterior thigh flap was still an option in this patient. However with this flap the patient would have had to be put in both supine and prone positions during surgery making the procedure more lengthy and difficult. It is also likely that two flaps would have had to be raised to cover the defect adequately.

While free flaps either latissimus dorsi, parascapular or even free anterior lateral thigh flaps were options, they are usually lengthy surgical procedures that are not routinely done in our hospital.

The pedicled anterior lateral thigh flap in our opinion was thus the best option for this patient. The flap has a long pedicle that allowed for adequate coverage of both groin areas. The flap also has good perforators once identified that could allow for an extensive flap of about 30 cm by 25 cm in dimensions [4]. In our patient the flap was about 25 cm by 18 cm in dimensions. The donor area can be closed primarily; however when not possible split thickness skin graft can be utilized to cover the defect.
